# Why did the dinosaurs become extinct? Could cholecalciferol (vitamin D_3_) deficiency be the answer?

**DOI:** 10.1017/jns.2019.7

**Published:** 2019-03-19

**Authors:** D. R. Fraser

**Affiliations:** Sydney School of Veterinary Science, Faculty of Science, The University of Sydney, Camperdown, NSW 2006, Australia

**Keywords:** Dinosaur extinction, Solar UVB radiation, Embryo mortality, Fossilised eggs, Mya, million years ago

## Abstract

Palaeontological deductions from the fossil remnants of extinct dinosaurs tell us much about their classification into species as well as about their physiological and behavioural characteristics. Geological evidence indicates that dinosaurs became extinct at the boundary between the Cretaceous and Paleogene eras, about 66 million years ago, at a time when there was worldwide environmental change resulting from the impact of a large celestial object with the Earth and/or from vast volcanic eruptions. However, apart from the presumption that climate change and interference with food supply contributed to their extinction, no biological mechanism has been suggested to explain why such a diverse range of terrestrial vertebrates ceased to exist. One of perhaps several contributing mechanisms comes by extrapolating from the physiology of the avian descendants of dinosaurs. This raises the possibility that cholecalciferol (vitamin D_3_) deficiency of developing embryos in dinosaur eggs could have caused their death before hatching, thus extinguishing the entire family of dinosaurs through failure to reproduce.

At the end of the Cretaceous era, about 66 million years ago (Mya), there was a mass extinction of many animal species around the world, including the large and diverse family of dinosaurs. The challenge of explaining this huge loss of biological diversity has been the province of geologists and palaeontologists. Their expertise in dating rocks and in reconstructing the characteristics of these long-gone life forms, from fossil bone and tooth fragments, is a remarkable achievement of scientific observation and analysis. Nevertheless, there has not been unanimous agreement in interpretations amongst these expert investigators. Because, from the vantage point of the present, the extinction of such a vast range of species seemed to have suddenly occurred, the concept has been proposed that some catastrophic environmental change is a unifying hypothesis to explain their disappearance. Some investigators, on the other hand, suggest that the time frame of the process of extinction from a biological viewpoint was very long and that the various species had declined in number over many generations. The conflict between catastrophism and gradualism to describe the process of extinction has been debated extensively during the second half of the 20th century.

The case for a catastrophic cause of mass extinction was strengthened by the discovery of a worldwide enrichment of the rare element iridium in a narrow band of geological strata formed 66 Mya^(^^1^^)^. The source of that iridium was proposed to be from a huge asteroid or comet that collided with the Earth, coinciding with the time of the mass extinctions^(^^2^^–^^4^^)^. The discovery of the Chicxulub impact crater in the Yucatan Peninsula on the Gulf of Mexico confirmed, from its size and structure, that a large celestial object had collided with the Earth at the time that the mass extinctions had occurred^(^^5^^)^. The consequences of that impact would indeed have been catastrophic, not just in the geographic region around the impact site, but worldwide because of environmental changes from an atmospheric dust and aerosol cloud that would have remained for years in the stratosphere^(^^2^^)^.

Those who favour a gradual decline towards extinction of all species of the dinosaur family, except the ancestors of modern birds, have some plausible arguments. The dating of fossils of all known dinosaur species is incomplete and is only well established for a range of dinosaurs in North America. It has been argued that long-term environmental changes had diminished the number of species over millions of years before the asteroid or comet impact, particularly the large-bodied Saurischian and Ornithischian dinosaurs^(^^6^^)^. Careful review of fossil records of both classes of dinosaurs has indicated that the number of species was in decline over millions of years. Thus the family Dinosauria would have been susceptible to extinction by a range of environmental changes to which they could not adapt^(^^7^^)^. Nevertheless, others have concluded that there is little evidence for a gradual decline worldwide in the diversity of dinosaur species, in contrast to that in North America, over the long term before the fossil record indicated that all non-avian dinosaurs had become extinct^(^^8^^)^.

Although the debate between the gradualists and the catastrophists appears to be still unresolved, both groups of palaeontologists do agree that the dating of dinosaur fossils ended in the era of the Chicxulub impact event. Even if the diversity of dinosaur species had been in decline, the gradualists do acknowledge that the worldwide environmental changes caused by that impact could have led to their final extinction.

Clearly the effect of the asteroid or comet collision would have been devastating to all life forms in a wide geographical region around the impact site. The proposed environmental changes that would have occurred worldwide include a period of dim sunlight because of solar radiation absorption by ejected particles and aerosols in the atmosphere and stratosphere. Consequently, there would have been a short-term temperature drop^(^^9^^)^, which, together with diminished sunlight, would have inhibited or killed photosynthetic plants. However, perhaps a more significant effect, as far as the dinosaurs were concerned, was a consequence of the geological site of impact. The Chicxulub site contains vast amounts of limestone (CaCO_3_), anhydrite (CaSO_4_) and gypsum (CaSO_4_.2H_2_O)^(^^10^^)^ as well as hydrocarbons^(^^11^^)^. A result of the asteroid impact would have been the ejection into the stratosphere of particulate carbon soot, CO_2_ and sulfate aerosols^(^^12^^)^. This would have had a marked cooling effect on the climate from the absorption of much solar radiation. Because such geological sites that are rich in sulfur and carbon deposits are not plentiful, it has been suggested that if the asteroid impact had occurred in most other regions of the world, the probability of mass extinctions would have been much less^(^^11^^)^.

Conclusions about the biological effects of the Chicxulub impact have focused on the undoubted environmental changes of temperature, light intensity, sea temperatures, sea levels and climate disruption in the months and probably years following the impact. The implications of these changes are that a consequent restricted food supply for many life forms that disappeared would have contributed to their extinction.

One result of the asteroid collision with Earth, that has not been considered from a biological perspective, is a particular effect from the ejection into the stratosphere of the sulfur aerosols. There are various calculations of the amount of sulfur, as sulfate or SO_2_, based on estimates of the size of the impacting asteroid and the density of sulfur deposits at the impact site. One estimate is that 326–527 gigatonnes of SO_2_ were distributed globally in the stratosphere^(^^13^^)^. Another, in broad agreement, put the quantity of stratospheric SO_2_ in the range of 200–400 gigatonnes^(^^10^^)^. The effect of such quantities of SO_2_ on global climate can be deduced from observations on the changes from sulfur ejected into the upper atmosphere from volcanic eruptions^(^^14^^)^. As an example, the Tambora eruption in Indonesia in 1815, the largest volcanic event in the past 200 years, produced only about 0·15 gigatonnes of stratospheric SO_2_. Yet in the following 12 months, this relatively small amount, compared with that from the Chicxulub impact, had a noticeable cooling effect on the world climate^(^^10^^)^.

An alternative environmental catastrophe postulated as the cause of dinosaur extinctions is a series of vast volcanic eruptions in India which created the Deccan basalt larval floods, known as the Deccan Traps. It has been argued that the timing of this volcanism is more likely to have been associated with the demise of the dinosaurs than the Chicxulub impact event^(^^15^^)^. Nevertheless, geological evidence puts the timing of the Deccan volcanism and the asteroid collision so close together that it has been suggested that the Chicxulub impact actually triggered the largest of the volcanic eruptions which gave rise to the Deccan Traps^(^^16^^)^. Although volcanic activity in general has not been considered to produce stratospheric sulfate aerosols in the quantities anywhere near that of the Chicxulub impact, the Deccan volcanism may have been an exception. Calculations from paleomagnetic measurements in a Deccan basalt escarpment identified single eruptive events, the largest of which could have released quantities of SO_2_ over years or decades, comparable with that released by the impact at Chicxulub^(^^17^^)^. Together, from volcanism and the asteroid collision, the SO_2_ emitted into the stratosphere could have had a prolonged climatic effect that would have lasted for years.

One biologically significant property of stratospheric SO_2_ is that it strongly absorbs solar UV light in the UVB wavelength range of 290 to 320 nm. In the steady atmospheric state of the present-day era, the intensity of UV light reaching the Earth's surface is attenuated by ozone^(^^18^^)^. However, the UVB-absorbing power of SO_2_ is 2·5 times greater than that of ozone^(^^19^^)^. The traces of SO_2_ from human activity such as that emitted by cities has a limited effect on UVB at ground level^(^^20^^)^. However, the mass of stratospheric sulfur from the Chicxulub impact, perhaps combined with that from the Deccan volcanism, would have been sufficient to completely block UVB reaching the Earth's surface for a decade or longer^(^^13^^)^.

One of the effects of UVB radiation is that it acts on 7-dehydrocholesterol in cells of the superficial integument of terrestrial animals to make, by a photochemical reaction, the pre-hormone, cholecalciferol. A characteristic of animals that depend on solar UVB radiation to supply cholecalciferol is that its precursor, 7-dehydrocholesterol, the penultimate metabolite in the synthesis of cholesterol, accumulates in superficial skin cells. In other cells, its concentration is very low because of rapid conversion to cholesterol. Cholecalciferol is more popularly known as vitamin D_3_ but under natural conditions it is not a nutrient. The food of most animals contains, at most, only traces of cholecalciferol. An adequate supply of cholecalciferol can therefore only be obtained by exposure of skin to UVB radiation from the sun. A deficiency of cholecalciferol causes a range of pathological changes, the most prominent of which is the bone disease of rickets or osteomalacia.

Much of the knowledge about the metabolism and function of cholecalciferol was obtained in the mid-20th century from studies of the domestic chicken (*Gallus domesticus*), a member of the avian descendants of the dinosaur family. Like the extinct dinosaurs, modern birds reproduce by depositing eggs encased in a hard shell of crystalline calcium carbonate. The egg shell not only protects the developing embryo inside, but it also is the source of Ca to mineralise the bony skeleton towards the end of the incubation period. Thinning of the egg shell from dissolution of calcium carbonate also weakens it, so that pecking by the beak of the chicken at full term fractures the shell, allowing the chicken to emerge.

Early in the development of knowledge about the biology of cholecalciferol, it was observed that hatchability of domestic hen eggs produced during winter was low, particularly if the laying hens had been kept indoors. Hatchability improved when hens were directly exposed to sunlight in spring^(^^21^^)^. It was therefore postulated that cholecalciferol, incorporated into yolk during egg production, was an essential requirement for avian embryonic development. When hens, reared away from sunlight, were fed a diet containing no cholecalciferol, the embryos in the eggs they produced died at 18–19 d of embryonic life, just before the 21-d scheduled time of hatching^(^^22^^)^. Likewise, the embryos in eggs from cholecalciferol-deficient turkey hens died at day 26–27 of their somewhat longer incubation period of 28 d^(^^23^^)^. Embryos in eggs from hens deprived of cholecalciferol developed hypocalcaemia and low bone mineralisation compared with embryos from hens fed a diet containing cholecalciferol^(^^24^^)^. From this pathology it was suggested that the embryos were unable to mobilise Ca from the shell and that the consequent hypocalcaemia caused muscle weakness which prevented the chicken from breaking the egg shell^(^^24^^,^^25^^)^.

The yolk of bird eggs, in contrast to other biological material, is quite rich in cholecalciferol. This is surprising because the yolk substances are derived from blood circulating through the ovary of the laying hen and the majority of cholecalciferol molecules in blood are in the form of its metabolite, 25-hydroxycholecalciferol, the precursor of the hormone, 1,25-dihydroxycholecalciferol. 25-Hydroxycholecalciferol has a long residence time in blood in comparison with its parent cholecalciferol which, after diffusing from its site of formation in the skin, is rapidly removed from blood by the liver. A specialised form of a cholecalciferol-binding protein transfers the trace cholecalciferol from blood into the developing yolk follicles in the ovary^(^^26^^)^.

In recent years there has been much interest in the many thousands of whole fossilised dinosaur eggs and egg shell fragments found around the world. Most attention has been directed at identifying the dinosaur species of each egg type and in drawing conclusions about the reproductive strategies from the arrangement of groups of eggs and of fossilised dinosaur embryos or juvenile dinosaurs that had died after hatching. In comparison with fossil dinosaur bones and teeth, little attention has been devoted to determining the age of the fossilised eggs and shell fragments. In his comprehensive book on dinosaur eggs, Carpenter^(^^27^^)^ catalogued the era of sites around the world where fossilised eggs had been discovered. There were twenty sites where eggs of the Jurassic era (130–80 Mya) were located and twenty-eight sites with eggs from the early Cretaceous era (100–130 Mya). However, most fossilised eggs have been dated from 187 sites of the late Cretaceous era (66–100 Mya). Despite considerable palaeontological study of the eggs, some containing fossilised embryos, the question does not seem to have been asked as to why so many eggs of this extinct family have survived for analysis over 66 million years. Why did so many whole eggs become fossilised? Were the eggs infertile or did the embryos die before hatching?

A study of *Hypselosaurus* dinosaur eggs from southern France and the Spanish Pyrenees concluded that shell thickness was abnormally thin in up to 90 % of the samples^(^^28^^)^. By comparison with egg biology of modern birds, the thinning of the shells was attributed to abnormal hormonal control of the calcification process, as a result of environmental stress. Strangely, as a possible explanation, there was no mention of cholecalciferol deficiency which is a well-established cause of thin shells in eggs of the domestic hen^(^^29^^)^.

Apart from shell thickness, another feature of dinosaur eggs that has been explored is the length of incubation. By remarkable deductive analysis it has been concluded that the incubation times of eggs of various dinosaur species are longer than those of their descendant modern-day birds. Lee^(^^30^^)^ estimated the metabolic rate of embryonic dinosaurs in comparison with that of birds and crocodiles and concluded that dinosaur egg incubation times were up to 76 d. However, Erickson *et al*.^(^^31^^)^, by measuring the growth lines in fossilised dinosaur embryo teeth estimated, more directly, the incubation times of dinosaur eggs. On the presumption that dinosaurs, like modern birds, were endothermic, they deduced that dinosaur egg incubation times were at least twice as long as those of birds, being up to 171 d, which is comparable with those of eggs of poikilothermic modern reptiles. Using this tooth growth-line technique, Varricchio *et al*.^(^^32^^)^ calculated that the incubation time of a theropod dinosaur (*Troodon formosus*) was 74 d compared with an average avian incubation time of 44 d.

Even if female dinosaurs had sufficient 25-hydroxycholecalciferol in their bodies to produce the hormone 1,25-dihydroxycholecalciferol to allow egg shell formation in the oviduct, unless they had a daily input of cholecalciferol to incorporate into the yolk being laid down in ovarian follicles, the eggs would have contained insufficient cholecalciferol to meet the needs of embryos during their prolonged period of incubation. The cholecalciferol-deficient dinosaur embryos, like those of the modern birds would either die during development, or else just before hatching. Although environmental changes leading to failure of food supply would certainly threaten the survival, over years or decades, of the wide variety of dinosaur species, the most effective mechanism of extinguishing the dinosaur family altogether would be one that prevented reproduction. One could speculate that if the modern bird descendants of theropod dinosaurs were today unable to obtain cholecalciferol from their environment, then over a short period of time they too would become extinct from reproductive failure.

Of course, if reproductive failure because of cholecalciferol deficiency was the cause of all dinosaur species becoming extinct, why then did other terrestrial vertebrates survive and evolve into the animals we know today? It is apparent that modern birds do not have a storage mechanism for cholecalciferol. Could other animals, such as mammals, have developed a means of conserving cholecalciferol to allow its many functions to continue when the environmental supply ceased? Present-day nocturnal mammals which seldom are exposed to solar UVB radiation seem to be able to maintain the functions of cholecalciferol without any apparent input from its formation in skin. There is evidence that the ancestors of modern mammals at the time of the dinosaurs had a nocturnal lifestyle^(^^33^^)^. By being both viviparous and nocturnal they may have avoided the reproductive failure of egg-laying dinosaurs. It is also possible that some dinosaur species survived the catastrophic events that were associated with most dinosaur extinctions. A few species that lived within the Antarctic Circle had anatomical features that suggested they were able to see in the dim light of winter and may have died out long after other Dinosauria had become extinct^(^^34^^)^. Apart from being able to see in the darkness of the Antarctic winter and accommodate to a cooling climate from the asteroid impact and volcanic eruptions, these polar dinosaurs may have also been able to conserve sufficient cholecalciferol, produced during the Antarctic summer, to maintain their capability for oviparous reproduction.

Would it be possible to test a cholecalciferol-deficiency hypothesis by further study of fossilised dinosaur eggs and embryos? Evidence could come from determining whether fossilised dinosaur embryos had bone pathology comparable with that, for example, of cholecalciferol-deficient turkey embryos that had died before hatching. Likewise, this theoretical cause of complete elimination of the dinosaur family would be supported if fossilised dinosaur egg shells, dated more exactly to the era of extinction, had the defective structure of egg shells produced by cholecalciferol-deficient birds.
